# Engraftment of Insulin-Producing Cells from Porcine Islets in Non-Immune-Suppressed Rats or Nonhuman Primates Transplanted Previously with Embryonic Pig Pancreas

**DOI:** 10.1155/2011/261352

**Published:** 2011-09-28

**Authors:** Marc R. Hammerman

**Affiliations:** George M. O'Brien Center for Kidney Disease Research, Departments of Medicine, and Cell Biology and Physiology, The Washington University School of Medicine, St. Louis, MO 63110, USA

## Abstract

Transplantation therapy for diabetes is limited by unavailability of donor organs and outcomes complicated by immunosuppressive drug toxicity. Xenotransplantation is a strategy to overcome supply problems. Implantation of tissue obtained early during embryogenesis is a way to reduce transplant immunogenicity. Insulin-producing cells originating from embryonic pig pancreas obtained very early following pancreatic primordium formation (embryonic day 28 (E28)) engraft long-term in non-immune, suppressed diabetic rats or rhesus macaques. Morphologically, similar cells originating from adult porcine islets of Langerhans (islets) engraft in non-immune-suppressed rats or rhesus macaques previously transplanted with E28 pig pancreatic primordia. Our data are consistent with induction of tolerance to an endocrine cell component of porcine islets induced by previous transplantation of embryonic pig pancreas, a novel finding we designate organogenetic tolerance. The potential exists for its use to enable the use of pigs as islet cell donors for humans with no immune suppression requirement.

## 1. Introduction

We have reviewed previously, for *J. Transplantation*, why transplantation of embryonic pancreatic primordia to replace endocrine pancreas function is advantageous relative to transplantation of either pluripotent embryonic stem (ES) cells, or of terminally differentiated (adult) organs [1]: (1) unlike ES cells, pancreatic primordia differentiate along defined lines without a need to steer differentiation; (2) there is no risk of teratoma formation; (3) the growth potential of cells within embryonic pancreas is enhanced relative to those in the terminally differentiated organ; (4) the cellular immune response to transplanted embryonic pancreas is attenuated relative to that directed against the adult organ; (5) the ability of avascular primordia to attract a host blood supply renders them less susceptible to humoral rejection than is donor-vascularized adult pancreas after transplantation across a discordant xenogeneic barrier; and (6) exocrine pancreatic tissue does not differentiate following transplantation of embryonic pancreas, obviating inflammatory complications that result from exocrine components. 

Transplantation of human embryonic pancreas in human hosts has been contemplated [2]. However, we [3–8] and others [9–12] have focused on the use of embryonic pancreas from the pig, a physiologically suitable donor for humans [13, 14]. Glucose tolerance can be normalized in streptozotocin- (STZ-) diabetic (type 1) LEW [3, 4, 7] rats or ZDF (type 2) diabetic rats [5] within 4 weeks following transplantation in mesentery of pig pancreatic primordia obtained very early during embryogenesis (on embryonic day 28 (E28)—just after the organ differentiates and prior to the time dorsal and ventral anlagen fuse) without host immune suppression. Rats are rendered permanently independent of a requirement for exogenous insulin to maintain normoglycemia. No circulating rat insulin can be detected in STZ-treated rats. Rather, porcine insulin circulates aftertransplantation of E28 pig pancreatic primordia levels of which increase after a glucose load. Cells with beta cell morphology expressing insulin and porcine proinsulin mRNA engraft in host mesentery, mesenteric lymph nodes, liver, and pancreas aftertransplantation. 

Cells originating from E28 pig pancreatic primordia transplanted in mesentery engraft similarly in non-immune-suppressed STZ-diabetic rhesus macaques [6, 8]. Glucose tolerance can be nearly normalized in non-immune-suppressed diabetic macaques following transplantation of E28 pig pancreatic primordia [1]. Porcine insulin, but not primate insulin, circulates after transplantation in macaques [6]. Exogenous insulin requirements are reduced in transplanted macaques [6]. Animals have been weaned off insulin for short periods of time, but not permanently [1]. The most likely explanation for the differential success between rats and macaques is that macaques weigh 20 times as much as rats. A STZ-diabetic rat can be rendered normoglycemic lifelong with no exogenous insulin requirement by transplantation of 5–8 pig pancreatic primordia. Extrapolating, it would take 100–200 primordia to render a diabetic macaque independent of exogenous insulin. This would require the sacrifice of about 10–20 pregnant sows and multiple surgeries with the attendant complications [7]. 

In lieu of increasing the numbers of transplanted primordia or transplant surgeries in diabetic rhesus macaques, we embarked on a series of experiments to determine whether porcine islets, a more easily obtainable and possibly more robust source of insulin-producing cells, could be substituted for animals in which embryonic pig pancreas already had been engrafted. To this end, we implanted adult porcine islets beneath the capsule of one kidney of rats or macaques, that several weeks earlier had been transplanted with E28 pig pancreatic primordia in mesentery. We employed the renal subcapsular site for islet implantation so that we could differentiate engrafted porcine tissue originating from the islets from tissue originating from prior mesenteric E28 pig pancreatic transplants, that never engraft in host kidney [7, 8]. In this setting, the contralateral (nontransplanted) kidney served as a control, as did kidneys from rats or macaques implanted with islets without prior transplantation of E28 pig pancreatic primordia in mesentery [7, 8]. As in experiments demonstrating engraftment of cells originating from E28 pig pancreatic primordia transplanted in mesentery of rats or macaques [3–6], we employed multiple are techniques to ascertain whether cells from porcine islets engrafted in kidney: immune histochemistry for insulin; in situ hybridization specific for porcine proinsulin mRNA; fluorescent in situ hybridization for pig X chromosomes; RT-PCR specific for porcine proinsulin mRNA; measurement of glucose-stimulated insulin release in vitro from implanted kidney tissue; electron microscopy [7, 8]. 

**Figure 1:**
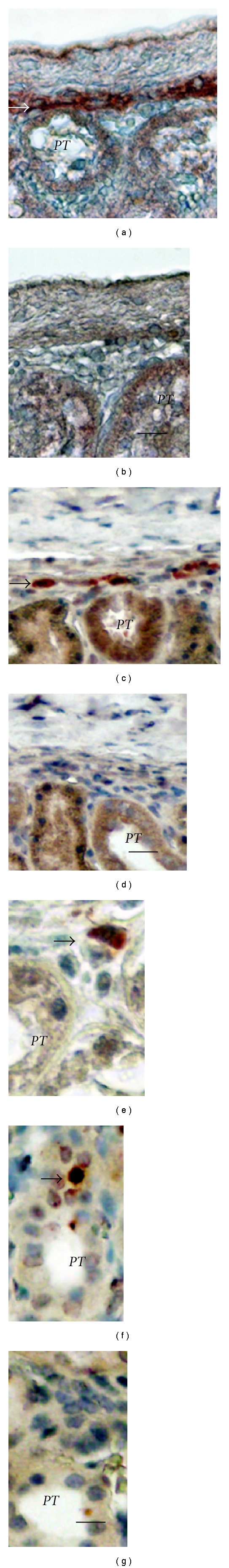
Sections of the islet-implanted kidney from a rat (a, b) or rhesus macaque (c–g) transplanted with E28 pig pancreatic primordia in mesentery followed by porcine islets in the renal subcapsular space stained using anti-insulin antibodies (a, c, and e) or control antiserum (b, d); or hybridized to an antisense (f) or sense (g) probe for porcine proinsulin mRNA. PT: proximal tubule. Arrows: positively staining cells (a, c, and e). Scale bars 10 um (a, b); 15 um (c, d), 7.5 um (e–g). Reproduced with permission from the American Society for Investigative Pathology [7] and from Organogenesis [8].

 Figures 1(a) and 1(b) show sections from a kidney of a STZ-diabetic rat implanted with porcine islets following transplantation of E28 pig pancreatic primordia in mesentery. Sections are stained using anti-insulin antibodies (Figure 1(a)) or control serum (Figure 1(b)). Cells that stain for insulin (Figure 1(a), arrows), but not with control serum (Figure 1(b)), are present in an expanded renal subcapsular space [7]. Figures 1(c)–1(g) show sections from a kidney of a STZ-diabetic rhesus macaque following transplantation of E28 pig pancreatic primordia in mesentery and subsequent implantation of islets in the kidney. Sections are stained using anti-insulin antibodies (Figures 1(c) and 1(e)) or control serum (Figure 1(d)) or hybridized to an antisense (Figure 1(f)) or sense (Figure 1(g)) probe specific [6] for porcine proinsulin mRNA. As was the case in rats (Figure 1(a)), a row of cells that stain for insulin is present in the subcapsular space (Figure 1(c) arrow). A high-power view of a single insulin-staining cell is shown in Figure 1(e) (arrow). It is polygonal with a round nucleus, a beta cell morphology [8]. No staining for insulin is observed in sections incubated with control antiserum (Figure 1(d)). A cell in the subcapsular space to which the antisense porcine proinsulin mRNA probe binds is shown in Figure 1(f) (arrow). No hybridization is observed if a sense probe is substituted for the antisense probe (Figure 1(g)). 

Neither cells that stain for insulin nor cells to which the probe for porcine proinsulin mRNA binds are present in contralateral (nonimplanted) kidneys of STZ diabetic rats [7] or macaques [8] in which E28 pig pancreatic primordia were transplanted previously in mesentery or in kidneys from STZ-diabetic rats [7] or macaques [8] into which porcine islets are implanted without prior transplantation of E28 pig pancreatic primordia in mesentery. Presumably, the implanted tissue is rejected by the host [7, 8].

**Figure 2:**
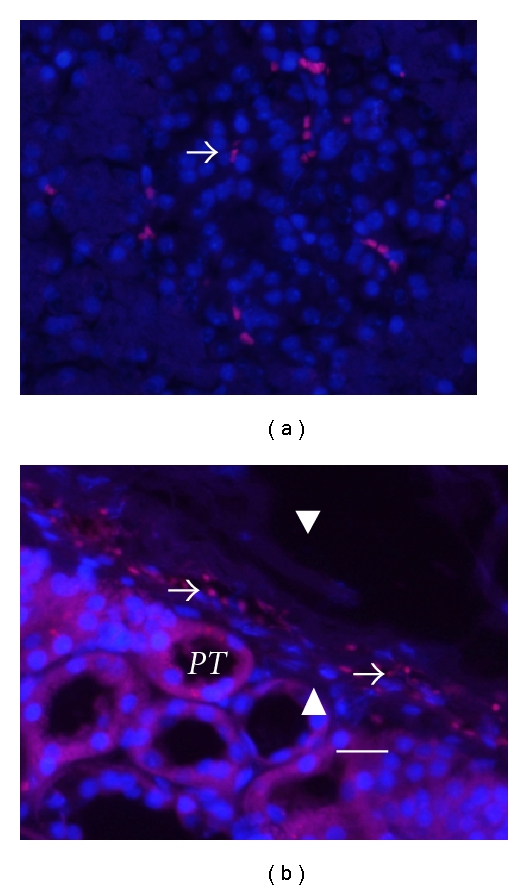
Fluorescence microscopy of tissue sections originating from (a) a normal porcine pancreas or (b) a subcapsular section from a kidney of a rhesus macaque transplanted with embryonic pig pancreas in mesentery and subsequently with porcine islets in that kidney. *PT*: proximal tubule, arrows: delineate pig X chromosomes. Arrowheads: renal capsule (b). Scale bar 10 um. Reproduced with permission from Organogenesis [8].

To provide additional evidence that cells in the kidneys of islet-implanted rats or macaques previously transplanted with E28 pig pancreatic primordia in mesentery are of porcine origin, we demonstrated using fluorescent in situ hybridization, that the cells contain pig X chromosomes [7, 8]. Shown in Figure 2(a) are pig X chromosomes in nuclei of cells from a normal porcine pancreas (positive control).  Figure 2(b) shows pig X chromosomes (arrows) in the nuclei of cells in the renal subcapsular space (arrowheads) from a STZ diabetic rhesus macaque transplanted with E28 pig pancreatic primordia in mesentery followed by porcine islets in kidney.

**Figure 3:**
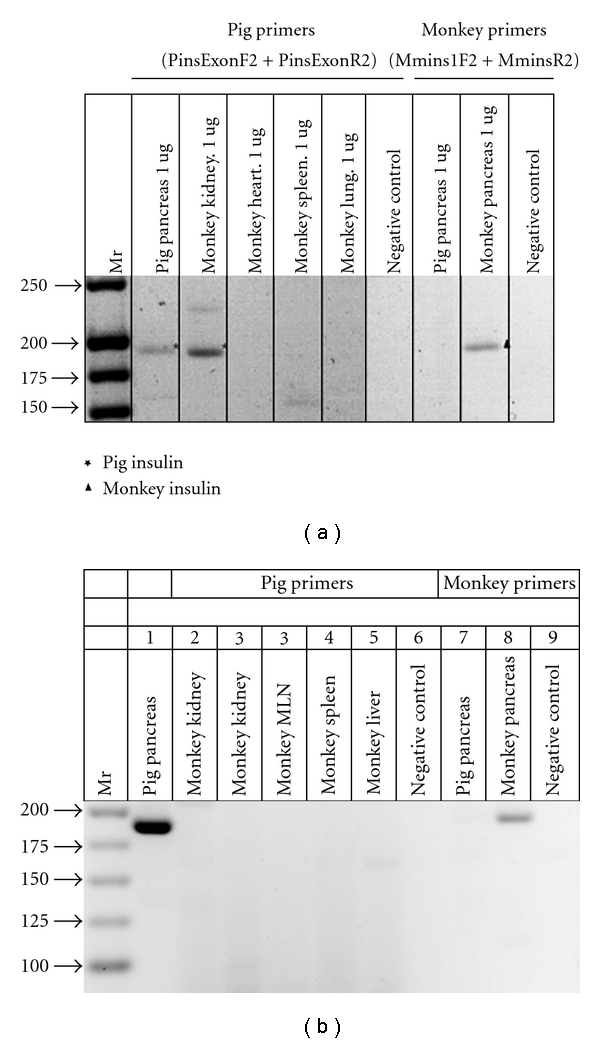
RT-PCR: (a) shown left to right are DNA molecular weights (Mr); amplification of bands using primers specific for porcine proinsulin from 1 ug RNA extracted from pig pancreas or from a rhesus macaque (monkey) transplanted with E28 pig pancreatic primordia in mesentery followed by implantation of porcine islets in the renal subcapsular space: kidney, heart, spleen, lung, a negative control for porcine-specific primers (no RNA); amplification of bands using primers specific for monkey proinsulin from 1 ug of pig pancreas RNA; monkey pancreas; a second negative control for macaque-specific primers. (b) Shown left to right are DNA molecular weights (Mr); amplification of bands using primers specific for porcine proinsulin from 2 ug RNA extracted from pig pancreas or from a rhesus macaque (monkey) implanted with porcine islets in the renal subcapsular space with no previous transplantation of E28 pig pancreatic primordia: kidneys, mesenteric lymph node (MLN) spleen, liver, a negative control for porcine-specific primers (no RNA); amplification of bands using primers specific for monkey proinsulin from 2 ug of pig pancreas RNA; monkey pancreas and a second negative control for macaque-specific primers. Pig primers amplify a 193 bps band. Monkey primers amplify a 199 bps band. Reproduced with permission from Organogenesis [8].

Multiple organs were excised from a STZ-diabetic macaque transplanted with E28 pig pancreatic primordia in mesentery and subsequently with porcine islets in the renal subcapsular space of one kidney. Tissues were homogenized individually and total RNA was purified. RT-PCR was performed using primers specific for pig or monkey proinsulin mRNA. Products were separated by electrophoresis on 3% agarose gels and their identities confirmed by sequencing in the Washington University Core Protein and Nucleic Acid Chemistry Laboratory [8]. Results are shown in Figure 3(a). The pig primers amplify a band of 193 bps in RNA originating from pig pancreas, corresponding to pig proinsulin insulin mRNA. The rhesus macaque (monkey) primers amplify a band of 199 bps corresponding to monkey proinsulin mRNA in monkey pancreas. Pig proinsulin mRNA is also detected in the islet-implanted monkey kidney. Multiple organs were excised from a STZ-diabetic macaque transplanted with porcine islets in the renal subcapsular space of one kidney with no prior transplantation of E28 pig pancreatic primordia in mesentery and RT-PCR performed as above. As shown in Figure 3(b), no pig proinsulin mRNA was detected in any monkey organ including the transplanted kidney.

To ascertain whether cells originating from kidney-implanted porcine islets function in rats or rhesus macaques, we determined whether the glucose tolerance of STZ-diabetic animals normalized partially by prior transplantation of E28 pig pancreatic primordia in mesentery was rendered normal by subsequent islet implantation, and measured glucose-stimulated insulin release from islet-implanted kidneys in vitro. Rats were rendered fully glucose tolerant by subsequent implantation of porcine islets in one kidney [7]. The glucose tolerance of macaques normalized partially by prior transplantation of E28 pig pancreatic primordia in mesentery was not improved by subsequent implantation of islets in kidney [8]. However, a rapid release of insulin by macaque kidney slices was demonstrated in vitro in response to elevation of glucose levels across the threshold for insulin release [8]. 

**Figure 4:**
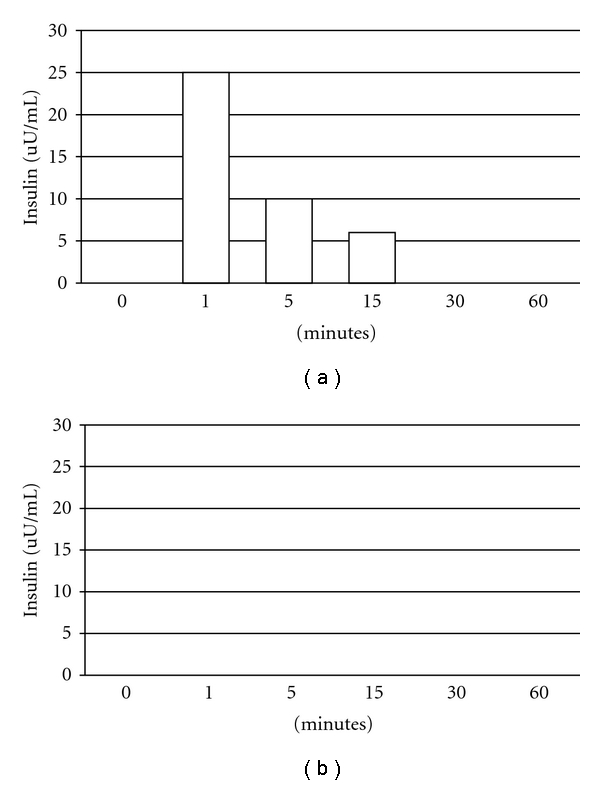
Levels of insulin (uU/mL) measured over 60 min in vitro after addition of glucose to tissue following time 0: (a) from a macaque kidney implanted with porcine islets following transplantation of E28 pig pancreatic primordia in mesentery; (b) the contralateral nonimplanted kidney. Reproduced with permission from Organogenesis [8].

As illustrated in a representative of 3 experiments using weight-matched tissue, no insulin could be detected at time 0 in supernatants from the implanted macaque kidney (Figure 4(a)). However, insulin was detectable by 1 min after increasing the glucose level in vitro. No insulin was detected at any time in any supernatants from the nonimplanted kidney (Figure 4(b)), or in supernatants from a kidney of a macaque in which porcine islets were implanted without prior transplantation of E28 pig pancreatic primordia in mesentery [8].

**Figure 5:**
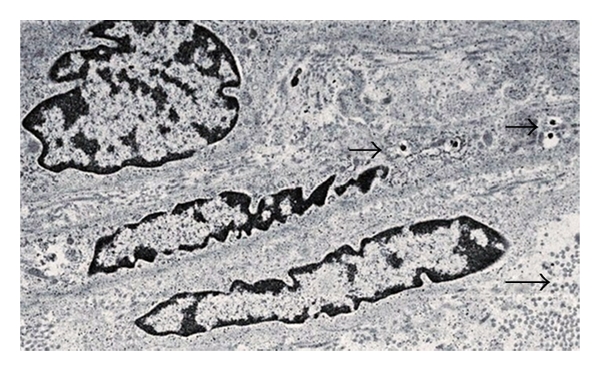
Electron micrograph of rhesus macaque kidney following sequential transplantation of E28 pig pancreatic primordia in mesentery and implantation of porcine islets in the kidney. Arrows: endocrine granules. Scale bar a 2 um. Reproduced with permission from Organogenesis [8].

Cells containing endocrine granules in an expanded renal subcapsular space were identified in electron micrographs of kidneys from rats implanted with porcine islets following transplantation of E28 pig pancreatic primordia in mesentery [7].  Figure 5 is an electron micrograph of the subcapsular space from a rhesus macaque kidney into which porcine islets were implanted following transplantation of E28 pig pancreatic primordia in mesentery. Shown is a cell with encapsulated granules (arrows) characteristic of endocrine secretory granules [8]. 

## 2. Discussion

The shortage of human pancreas donor organs imposes severe restrictions on the use of allotransplantation to treat diabetes mellitus [15–22]. When performed, whole pancreas transplantation requires use of potent immunosuppressive medications that have significant complications. Newer, more targeted immunosuppressive regimens that do not require steroids or high-dose calcineurin inhibitors make islet transplantation a more attractive option. However, side effects of immune suppression that must be maintained so long as the islet graft functions remain a source of morbidity and even mortality [15]. Thus transplantation therapy for diabetes trades one set of morbidities (associated with diabetes and its medical treatment) for another (associated with immune suppression). 

The severity of humoral rejection effectively precludes the use of pigs as whole pancreas organ donors for humans. However, because they are vascularized by the host posttransplantation, islets like other cell transplants are not subject to humoral rejection. Porcine islets are rejected within two weeks of transplantation in non-immune suppressed non-human primates [8, 16–18]. Experience with pig to primate islet or neonatal islet transplantation in immune suppressed non-human primates shows that sustained insulin independence can be achieved, but only through the use of agents that are not approved for human use or that result in a high level of morbidity and mortality [19–21]. Thus, the need for host immune suppression is a barrier for pig-to-human islet xenotransplantation. 

Xenotransplantation of embryonic pig pancreatic primordia in lieu of mature pig organs or porcine islets couples the wide availability of pig organs with the immunological advantages inherent in transplanting cellular embryonic tissue, circumventing humoral rejection and obviating the need for host immune suppression [1]. However, obtaining embryonic pig pancreata is technically challenging because surgery must be performed on multiple pregnant sows and isolation carried out from scores of embryos to obtain sufficient numbers of primordia. Furthermore, transplanting pancreatic primordia in mesentery of primates is invasive, requiring that a host laparotomy be performed on one or more occasions. In contrast, porcine islets can be isolated in large quantities from a single pig pancreas, and infusion of porcine islets can be carried out via the portal vein infusion without a laparotomy [22].

Cells from porcine islets do not survive afterimplantation in rat or macaque kidneys without prior transplantation of E28 pig pancreatic primordia transplantation in mesentery [7, 8]. Whole porcine islets do not engraft in kidneys. Rather, an endocrine (beta cell) component originating from porcine islets does so [7, 8]. Ours is the first report describing sustained survival of such cells following transplantation of porcine islets in non-immune-suppressed primates. Glucose tolerance in diabetic rats not fully normalized by prior transplantation of E28 pig pancreatic primordia in mesentery is corrected following subsequent implantation of porcine islets in kidney [7]. In contrast, correction is not observed following E28 pig pancreatic primordia transplantation and porcine islets implantation in macaques [8]; that is, consistent with previous observations that glucose tolerance is more difficult to correct in macaques than in rats [6]. It is possible that the cell component, although of sufficient mass to normalize glucose tolerance in diabetic rats following implantation of islets [7], is insufficient following implantation in rhesus macaques [8]. Implantation of more islets (isolated from more than one adult pig pancreas) in kidney or infusion of porcine islets into a site from which insulin can act more directly on liver (the portal vein) [22] following transplantation of E28 pig pancreatic primordia in mesentery may be a better way to normalize glucose tolerance. Alternatively, it may be that the mass of engrafted cells originating from porcine islets implanted in kidney (perhaps derived from a numerically small stem cell component within the islets) [7, 8] is insufficient to impact on control of circulating glucose in an animal as large as a macaque.

Schroeder et al. [23] define transplantation tolerance as immune unresponsiveness to the transplanted organ, but not to other antigens in the absence of ongoing immunosuppression. Lewis rats transplanted with E28 pig pancreatic primordia retain reactivity to other porcine xenoantigens (E28 pig renal primordia are rejected [4]). Thus, our findings are consistent with induction of specific tolerance [23] to a cell component (either beta cells or a stem cell component that differentiates into insulin-producing cells) of adult porcine islets implanted in Lewis rats by previous transplantation of E28 pig pancreatic primordia.

Engraftment of pancreatic progenitors transplanted across a xenogeneic barrier to non-immune-suppressed immune-sufficient hosts has been reported twice previously. Eloy et al. described normalization of glucose posttransplantation of E15, but not E18 embryonic chick pancreas into liver of non-immune-suppressed STZ-diabetic rats [24]. Abraham et al. [25] described successful xenoengraftment in multiple organs of human pancreatic islet-derived progenitor cells infused in nonimmunosuppressed mice. Neither Eloy et al. [24] and Abraham et al. [25] nor we [3–8] define an immunological mechanism for the finding. Although the antigenicity of fetal tissues may be less than that of corresponding adult tissues, animal data suggest the reduction is not enough by itself to ensure permanent graft survival [26]. Thus, the use of embryonic tissue (pancreas) per se cannot explain the results.

Host immune suppression is required for successful engraftment of embryonic pig pancreas in rodents [11] or non-human primates [12] carried out using methodology alternative to ours. Therefore, it is likely that one or more factors in the methodology we employ [3–5, 7], different from that used by others (the protocol of Tchorsh-Yutsis et al.) [11], are critical for engraftment without an immune suppression requirement. Such factors include those listed in Table 1 for studies that employ rats as hosts for pancreatic primordia. First, the developmental stage of donor pig embryos from which primordia are obtained impacts on the host immune response. We have shown that E35 pig pancreatic primordia are rejected in Lewis rats following transplantation, employing conditions under which E28 or E29 pig pancreatic primordia are engrafted [4]. While we have no experience transplanting E42 pig pancreatic primordia in rats, the preferred stage for studies described by Tchorsh-Yutsis et al. [11], we would expect them to reject based on our experience with E35 pancreatic primordia [4]. Second, it is likely that that incubation of embryonic pancreas prior to transplantation with one or more growth factors and cytokines (iron-saturated transferrin; prostaglandin E1 and vascular endothelial growth factor; hepatocyte growth factor) [3–5, 7] alters the host immune response. Tchorsh-Yutsis et al. do not employ such agents [11]. Third, it is possible that hyperglycemia in diabetic hosts [3–5, 7] impairs the immune response to embryonic pancreas. Tchorsh-Yutsis et al. transplant pancreatic primordia into nondiabetic rats [11]. Fourth, the transplantation site and technique probably impacts on the host immune response. We interpose pancreatic primordia between sheets of mesentery [3–5, 7]. Tchorsh-Yutsis et al. transplant into pockets of omentum and secure using suture [11], the latter in itself likely to trigger inflammation. We have proposed [27] that transplantation of E28 pig pancreatic primordia in the mesentery and migration of cells to mesenteric lymph nodes and liver recapitulates events that occur during induction of oral tolerance [28–30], which is dependent on antigen transport via afferent lymphatics into the draining mesenteric lymph nodes [30]. In effect, we suggest that heterotopic introduction of embryonic pig pancreas in rat or primate mesentery coopts the function of the gut-associated lymphoid tissues (GALT), a complex, redundant [28–30], and phylogenetically ancient system [31, 32] of which embryonic pancreas is a part [33], that, under normal conditions, induces peripheral tolerance to ingested antigens in jawed vertebrates and their descendants.

Interestingly, GALT may have served similarly to prevent an immune response to insulin-producing cells scattered originally in the gut epithelium of primitive vertebrates [31, 32] and has been proposed to induce tolerance or immune suppression towards islet cell antigens during normal embryonic development [33]. Developmentally controlled lymphogenesis establishes a preferential trafficking route from the gut to pancreatic lymph nodes, a GALT component, in which T cells can be activated by antigens drained from the peritoneum and the gastrointestinal tract. Intestinal stress modifies the presentation of pancreatic self-antigens in pancreatic lymph nodes. The convergence of endocrine and intestinal contents at this site may explain the link between an autoimmune pathogenesis for type 1 diabetes and environmental provocation [33, 34]. Low doses of orally administered antigen induce antigen-specific peripheral tolerance through active suppression of T cells and induction of clonal anergy. High doses induce tolerance by extrathymic deletion of antigen-reactive T cells [35]. It was proposed originally that oral tolerance depends exclusively on antigen uptake by cells within intestinal Peyer's Patches [30]. However, recently it has been shown that high-dose oral tolerance can be induced in the absence of Peyer's Patches so long as mesenteric lymph nodes are present [30, 36]. 

Harada et al. have proposed a similar coopting of oral tolerance to explain the muted immune response in vivo and by cells from mesenteric lymph nodes in vitro to a colon carcinoma of BALB/c origin or a human CD80-transfected DBA/2 mastocytoma injected into the subserosal space of cecum in BALB/c mice relative to tumors injected subcutaneously [37]. 

One way to confirm a causative link between gut immunity and our ability to transplant E28 pig pancreatic primordia and porcine islets in non-immune-suppressed hosts would be to “break” the established oral tolerance [38], using glucose control as a readout in the rat mode, theoretically possible so long as extrathymic deletion of antigen-reactive T cells [35] has not occurred. 

In any case, we have demonstrated in two species [7, 8], a novel finding that prior transplantation of embryonic tissue (pancreas) enables engraftment of a cell component from differentiated adult tissue from the same organ (islets) transplanted subsequently, without the need for host immune suppression, a phenomenon, the immunologic mechanism for which remains undefined, that we term organogenetic tolerance [27]. Applicability of the finding to organ replacement therapy in humans awaits definition. The potential exists for its use to enable the use of pigs as islet cell donors for humans with a need for no immune suppression.

## Figures and Tables

**Table 1:** Methodology employed for transplantation of embryonic pig pancreas in immune sufficient non-immune-suppressed rats.

	References [3–5, 7]	Reference [11]
Developmental stage of donors	E28 preferred, E29	E28, E42 preferred
Incubation prior to transplantation	Growth factors & cytokines	None
Diabetic status of the host	Diabetic	Nondiabetic
Transplantation site	Interposed within mesentery	Sutured in omentum
